# Life-History Traits of a Small Cosmopolitan Copepod (*Oithona similis*) in the Barents Sea: A Review

**DOI:** 10.3390/biology15010027

**Published:** 2025-12-23

**Authors:** Vladimir G. Dvoretsky, Alexander G. Dvoretsky

**Affiliations:** Murmansk Marine Biological Institute of the Russian Academy of Sciences (MMBI RAS), 183038 Murmansk, Russia

**Keywords:** *Oithona similis*, Barents Sea, abundance, production, morphological variables, life cycle, populations, environmental drivers

## Abstract

This review synthesizes current knowledge on the biology and ecology of the small cyclopoid copepod *Oithona similis*, a prevalent planktonic species in the Barents Sea, during the period of Arctic warming since the early 2000s. The region serves as an effective model system for examining the influence of different water masses on Arctic zooplankton dynamics. The highest abundances and biomass of *Oithona similis* are observed in Murmansk Coastal Waters (MCW) and Arctic Waters (ArW). Although its contribution to total zooplankton biomass is generally lower than that of higher copepod taxa, it can account for up to 27–35% seasonally and regionally. Ovigerous females are most abundant in Novaya Zemlya Waters (NZW) and ArW. Egg production rates exhibit a decreasing trend from south to north across the sea. Morphometric analyses reveal an increase in prosome length for both sexes, while relative antenna size diminishes from the south (MCW) to the north (ArW). The highest mortality rates occur during summer, coinciding with peak abundances of *Oithona similis*, its predators, and parasites, as well as increased interspecific competition. Based on morphological and reproductive parameters, three distinct populations are delineated within the Barents Sea: southern (MCW), central (Atlantic Water/Barents Sea Water), and northern/eastern (ArW/NZW), with respective life cycle durations of 11–12, 9–10, and 11 months, and typically one to two generations per year. The primary environmental drivers influencing population abundance, biomass, size, and reproduction are temperature and salinity, while chlorophyll *a* concentration predominantly affects mortality rates.

## Simple Summary

This review highlights the important role of the small copepod *Oithona similis* in the Arctic ecosystem, particularly in the Barents Sea amid ongoing warming. *Oithona similis* is a common planktonic species whose abundance and biomass vary across different water masses, with notable populations in coastal and Arctic waters. Despite contributing less to overall zooplankton biomass compared to other copepods, it can still represent a significant portion during certain seasons. Its reproductive activity and morphological characteristics differ regionally, influenced by environmental factors such as temperature, salinity, and chlorophyll levels. The species exhibits distinct populations across the southern, central, and northern parts of the sea, each with unique life cycle durations and reproductive patterns. The highest mortality rates occur in summer, driven by predation, parasitism, and competition. These findings underscore the species’ adaptability and the influence of environmental conditions on its biology. Understanding these dynamics is crucial, as *Oithona similis* plays a vital role in the Arctic food web, and its responses to climate change may have significant ecological implications in this rapidly changing region.

## 1. Introduction

The Barents Sea represents the largest Arctic continental shelf region. The total area of the sea is approximately 1,400,000 km^2^ with an average depth of 230 m [1]. The maximum depth (500 m) is registered in the western part of the Bear Island Trench [2]. The northern part of the Barents Sea ecosystem borders the Arctic Ocean, while the western, eastern, and southern boundaries are defined by the shelf break toward the Norwegian Sea, the Novaya Zemlya archipelago, and the coasts of Russia and Norway, respectively [3]. The Barents Sea is a transition zone for warm Atlantic waters entering from the Norwegian Sea and contributing to the deep-water areas of the Arctic Ocean [4,5]. In the northern part, the upper layer is occupied by Arctic waters with relatively low salinity and density partly originating from the Arctic Ocean [6]. Atlantic water is mainly confined to the southern, western, southwestern, and central parts [7]. The boundary between these two main water masses is referred to as the Polar Front [8]. It is located across the eastern slope of the Svalbard Bank and eastward from Hopen towards Novaya Zemlya [1,3].

Among all Arctic regions, the Barents Sea is considered the most productive area [9,10]. Here, phytoplankton produce 49% primary production of the total primary production on the pan-Arctic shelf [2,7,11]. Such high productivity is mainly utilized by plankton animals that are the main food resource for pelagic fish [12]. Subsequently, these pelagic fish, particularly capelin *Mallotus villosus* and herring *Clupea harengus*, are consumed by Atlantic cod *Gadus morhuaand* and haddock *Melanogrammus aeglefinus* [13,14]. For this reason, the Barents Sea includes one of the world’s largest fishing areas [15], which is located in permanently ice-free waters in the south and southwest regions [16] strongly influenced by warm waters from the Norwegian Sea [6].

**Figure 1:**
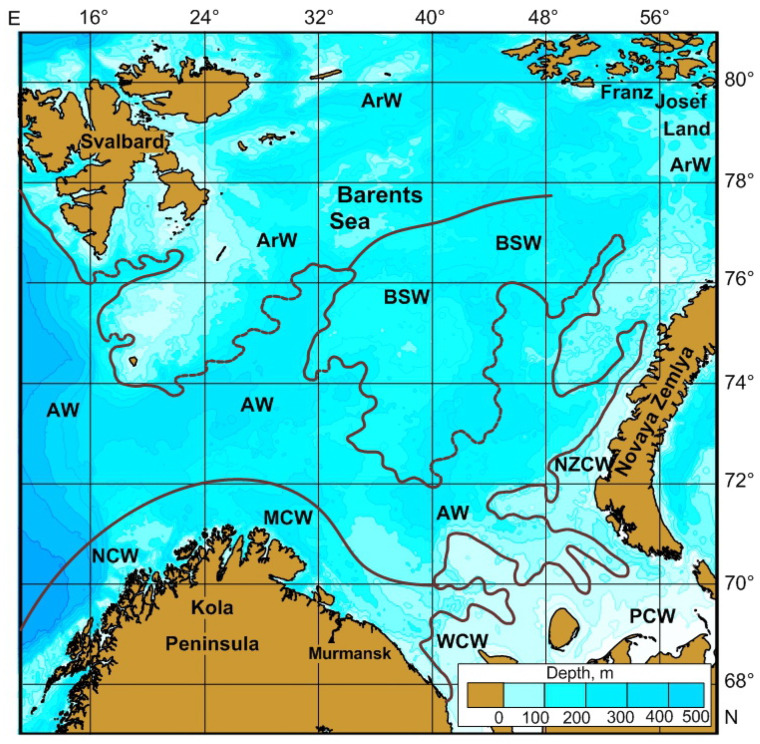
Barents Sea: location of main water masses [2,17,18]. MCW—Murmansk Coastal Water, AW—Atlantic Water, ArW—Arctic Water, BSW—Barents Sea Water, PCW—Pechora Coastal Water, NZCW—Novaya Zemlya Coastal Water.

There are six major water masses in the Barents Sea [2,17,18]: (a) Murmansk Coastal Water (MCW)—coastal areas of the Kola Peninsula and adjacent waters (southern part); temperature range 1–9 °C; salinity 33.8–34.7 psu; (b) Atlantic Water (AW)—central and western parts of the sea; temperature > 3 °C, salinity > 35.0 psu; (c) Arctic Water (ArW)—northern part of the sea (temperature < 0 °C, salinity 32.0–34.8 psu); (d) Barents Sea Water (BSW)—eastern part of the sea (temperature –1.5–+5 °C; salinity 34.5–35.0 psu); (e) Pechora Coastal Water (PCW)—south-eastern part = Pechora Sea (temperature –1.9–+8 °C; salinity 32.0–34.0 psu); (f) Novaya Zemlya Coastal Water (NZW)—eastern part (temperature –1.9–+8 °C; salinity 32.0–34.0 psu) (Figure 1).

Copepods are among the most frequent, abundant, and widely distributed marine organisms in the World’s Ocean [19,20,21]. These animals are a major food resource for ichthyoplankton, pelagic fishes, young demersal fishes and macrozooplankton [22,23,24,25]. Copepod assemblages link primary producers and microzooplankton to higher trophic levels [26] and therefore, play an important role in pelagic food webs worldwide [27,28]. The significant role of copepods in the functioning of the Barents Sea ecosystem makes this group a subject for extensive studies [29,30]. Most of the data on the biology of planktonic crustaceans are already available especially for larger taxa (e.g., *Pseudocalanus* and *Calanus* spp.) because they are the most important food resource for commercial fish [31,32,33]. However, results of recent studies suggest that small species may be more significant in marine food webs than that was previously considered [34,35,36].

The cyclopoid copepods of the genus *Oithona* occur in many marine geographical regions [37,38,39,40,41,42,43,44,45,46,47,48]. The population density and biomass as well as population structure of *Oithona* spp. demonstrate significant variations at interannual, seasonal, and regional scales [49,50,51,52]. Moreover, the cyclopoid copepod *Oithona similis* Claus, 1866 is a cosmopolitan species that inhabits circumglobally and it has been described as a eurythermal, euryhaline, and omnivorous species well adapted to a wide range of habitats [38]. It has been suggested that *O. similis* may be considered the most numerous copepod in the world [34]. *Oithona* spp. feed on smaller-sized organisms including heterotrophic or autotrophic microplankton, and copepod nauplii, and they are the preferred food of fish larvae and other zooplanktivores [53,54,55]. In the Barents Sea, *O. similis* represents an example of the genus *Oithona* that reaches maximum abundance among other small planktonic copepods [56,57,58,59,60,61,62]. *O. similis* appear to be less affected by seasonal changes in phytoplankton abundance than larger herbivorous copepods (*Calanus* spp. and *Pseudocalanus* spp.). This small opportunistic species remains active all year round and does not undergo diapause in Arctic regions [51,52,63,64,65,66,67].

The abundance and biomass of *Oithona similis* can, in certain seasons and regions of the Barents Sea, surpass those of *Calanus finmarchicus* [68]. Due to their high average abundance and small body size, *O. similis* plays a crucial role as a food source for various zooplankton taxa, including larger copepods, some macrozooplankton groups (such as chaetognaths, hyperiid amphipods, and medusae), as well as for fish larvae and planktivorous fish species, including capelin and herring. In cold years, when the biomass of *C. finmarchicus* declines relative to more typical or warmer years, *O. similis* may assume the role of the primary food source for adult fish in the Barents Sea [68]. While the biology of *O. similis* has been extensively studied in temperate waters [42,43,44,48,54,55,69,70,71], recent years have seen significant advances in understanding its ecology in the Barents Sea [57,58,61,62,72,73,74,75,76,77,78,79,80,81].

The Arctic environment has undergone substantial climatic changes over the past few decades [82,83]. Key factors driving significant shifts in pelagic ecosystems include rising water and air temperatures, retreating Arctic sea ice, declining sea-ice extent, alterations in oceanographic regimes, and intensified inflow of Atlantic Water [84,85]. A particularly notable phenomenon is the Atlantification of the Barents Sea, characterized by the northward expansion of boreal taxa and the concomitant migration of cold-water species toward the Arctic Ocean [86]. Additionally, warming trends have extended the growth season of phytoplankton assemblages [6,87], resulting in increased primary productivity and enhanced chlorophyll *a* concentrations in the Barents Sea [88]. These changes have cascading effects across all trophic levels, impacting both large zooplankton and smaller copepods [61,62,79,80,89].

This review synthesizes critical data on the biology of *Oithona similis* in the Barents Sea, aiming to evaluate the predominant environmental drivers affecting this species and enhance our understanding of its life history within the region. The review addresses key aspects including the distribution, abundance, biomass, reproductive strategies, mortality, morphology, and life cycle of *O. similis* during the recent period of Arctic warming that has characterized the early 21st century. The insights derived from this analysis may serve as a valuable resource for monitoring shifts in zooplankton assemblages in the Barents Sea in response to climatic perturbations, thereby contributing to a more comprehensive understanding of how pelagic ecosystems adapt to variations in environmental conditions.

## 2. Abundance and Biomass of *Oithona similis*

Population density of *O. similis* varies in a wide range depending on the location of sampling stations, water masses (environmental conditions), and season (Table 1).

In summer, the lowest values of *Oithona similis* abundance and biomass are generally observed in areas affected by freshwater discharge, such as the Pechora Sea and southern Kola Bay, where salinity is low and mean abundance does not exceed 100 individuals m^−3^ (ind. m^−3^). In contrast, maximum abundances are recorded in regions influenced by Arctic and Atlantic waters, particularly in frontal zones (AW–ArW, AW–BSW), as well as in coastal areas enriched by organic inputs from the shore, especially in bays near significant seabird colonies (MCW and NZW), where mean abundance can reach up to 1000 ind. m^−3^ [81,93]. Seasonal fluctuations in total *O. similis* abundance are closely linked to the location of water masses, with overall abundance typically increasing during the spring and summer, and exhibiting a marked decline in late winter.

During the summer months, *O. similis* constitutes 21–35% (with peaks up to 50%) of total mesozooplankton abundance in MCW, 35–45% (up to 75%) in AW, 23–48% in coastal waters off Novaya Zemlya, and 25–28% in ArW. Studies conducted in Kola Bay (coastal waters of the southwestern Barents Sea, MCW) indicate that the total abundance of *O. similis* varies from 109 to 256 ind. m^−3^ in winter, 120 to 2760 ind. m^−3^ in spring, 410 to 1300 ind. m^−3^ in summer, and 300 to 400 ind. m^−3^ in fall, with peak abundance recorded between 4700 and 9600 ind. m^−3^ during June and July [57,74]. In Kola Bay, this species comprised 15% of total abundance in spring, 25% in fall, and 35% in winter [74].

Overall, the total biomass of *O. similis* is considerably lower compared to larger copepods and other crustaceans, such as krill and hyperiids, in the Barents Sea [32,33]. The contribution of *O. similis* to the total zooplankton biomass exhibits considerable variability depending on the season, water mass, and specific year of study (Table 1). Notably, *O. similis* is absent in certain estuarine areas characterized by extremely low salinity, such as the delta of the Pechora River and the mouth of Kola Bay [58,90]. In other regions of the Barents Sea, the species’ proportion of total biomass ranges from 0.1% to 27.3% during summer (see Table 1). In specific seasons, such as winter 2010 in BSW, *O. similis* may account for as much as 35.4% of total zooplankton biomass [95,96]. Similar trends have been documented in the White Sea, where the relative biomass of *O. similis* fluctuated between 0.1% and 30% (mean 3.1%) during the summer periods of 2001 and 2008 [105]. In the Kara Sea, *O. similis* represented 0.2–11.1% of the total zooplankton biomass during winter [106], while accounting for 0.3–41.0% (mean 6.1%) in the summer of 2012 [107]. For comparison, in Godthåbsfjord (Greenland waters), the relative density of *Oithona* spp. (predominantly *O. similis*) peaked in summer, coinciding with maximum seasonal abundance [52]. There, the seasonal cycle of *Oithona* spp. density exhibited clear succession patterns, with low abundance recorded in late winter to early spring, surging to peak levels from July to August (3000–7330 ind. m^−3^, recalculated from original data by Zamora-Terol et al. [52]). Conversely, in Kongsfjorden (Svalbard waters), *O. similis* was present year-round, with maximum values observed during winter (November) and lowest abundances recorded in summer [63]. In Isfjorden, a pronounced peak in *O. similis* abundance was detected in September (5000–6500 ind. m^−3^), while the minimum occurred in March to April [108].

Environmental forcing significantly influences zooplankton communities across the World’s Oceans [2,7,11,19]. In the Barents Sea and other Arctic regions, the response of plankton assemblages is contingent upon regional, seasonal, and climatic conditions [33,51,52,65,75,98,100,109,110]. Within coastal waters (MCW), *Oithona similis* exhibits a strong negative correlation with salinity, while its abundance positively correlates with water temperature [57,58]. Conversely, in PCW, salinity positively affects both abundance and biomass, whereas temperature shows a negative correlation with these parameters. Interestingly, in AW, total abundance of *O. similis* was also negatively correlated with temperature [57,58]. This observation likely results from elevated water temperatures during the study periods, which are unfavorable for *O. similis*, as the species is adapted to colder conditions.

In Arctic regions, total abundance of *O. similis* generally increases as water temperature decreases; however, in some instances, no significant relationships were observed between environmental variables and indices of *O. similis* [57,58]. Similar trends have been noted in other research. For example, a comparison of long-term datasets from the North Atlantic and Mediterranean Sea revealed that *O. similis* abundance was negatively correlated with water temperature only in the Mediterranean, where the species exhibited an optimal temperature range of 15 to 20 °C—beyond which higher temperatures limited *O. similis* occurrence [70]. In the North Atlantic, the mean abundance of *O. similis* was lower than in the colder waters of the Barents Sea and adjacent Arctic regions. Notably, peak density of *O. similis* near Plymouth was recorded in March, with 286 ind. m^−3^ for females and 737 ind. m^−3^ for copepodites [48]. In Svalbard waters, sea water temperature was identified as the primary factor controlling 62% of the variance in *O. similis* abundance [108]. Latitudinal analyses indicate a preference for colder waters, suggesting ecological implications for studying the effects of climatic forcing on zooplankton assemblages and their phenology [70,71].

Food availability also influences *O. similis* abundance in the Arctic and other areas [42,53,54,55]. However, no correlations have been found between *O. similis* population density and phytoplankton biomass, as measured by chlorophyll *a* concentrations (Chl-a), in the Barents Sea [57]. Similarly, Castellani et al. [70] observed only a weak positive correlation between *O. similis* abundance and Chl-a in the North Atlantic and Mediterranean. This lack of correlation may stem from the feeding behavior of *O. similis*, which prefers to consume ciliates (e.g., *Strombidium* spp. and *Myrionecta* spp.) and other protozooplankton, although phytoplankton are not completely disregarded as a food source [42,53,54]. Seasonal lipid analyses of *O. similis* have indicated high levels of 18:1 (n-9) fatty acids throughout the year, suggesting a generally omnivorous, carnivorous, or detritivorous diet in the Arctic Kongsfjorden (Svalbard waters) [63].

Thus, the contribution of *Oithona similis* to total zooplankton abundance in the Barents Sea is substantial. However, its proportion in the total zooplankton biomass has been relatively low in the context of recent climatic changes in the Arctic. Temperature emerges as the primary factor influencing *O. similis* abundance in the Barents Sea. The species’ relative importance in zooplankton assemblages tends to diminish during warming periods compared to colder years, likely due to the unfavorable conditions associated with higher water temperatures.

## 3. Reproduction of *Oithona similis*

In summer, *Oithona similis* females comprised 45% of the total population abundance across the Barents Sea, with the exception of the northern regions (ArW and BSW). This proportion increased as one moved from the southern coastal waters (MCW) to the central Atlantic waters (AW), then decreased again in the northern regions (ArW) [57]. A similar trend was observed for males; however, copepodite IV exhibited a latitudinal decrease from MCW to AW, peaking in ArW. The sex ratio of *O. similis* varied between 1:3 and 1:8, averaging around 1:5. Notably, environmental factors did not influence the sex ratios, and inter-annual variations were minimal [57].

During the summer, ovigerous females (those carrying egg sacs) were found in all regions of the Barents Sea, with their relative abundance ranging from 5% (in AW) to 52% (also in AW) [73]. Mean values showed an increasing trend from the south (MCW and AW) to the north, with particularly high proportions in the northern shelf waters (NZW) and ArW (Table 2). Overall, lower numbers of ovigerous females were observed in southern coastal waters during the summer, indicating a spatial pattern in the reproductive behaviors of *O. similis* [73].

The female reproductive effort (FRE), defined as the ratio of female carbon mass to egg carbon mass, was found to be lowest in ArW, AW, and BSW in 2007 (25%). In contrast, maximum FRE values were recorded in MCW and AW in 2005, ranging from 45% to 47% [57,73]. Higher values of female reproductive effort were noted in MCW, whereas the northern regions exhibited consistently low FRE (Table 2).

A latitudinal trend in egg diameter was observed for *O. similis* in the Barents Sea, with the smallest diameters recorded in PCW and NZW, intermediate values in MCW and AW, and the largest diameters found in ArW and BSW. Egg diameters varied from 44 to 70 µm during the summer seasons [73]. Comparisons of mean egg diameter did not reveal significant differences between summer and autumn periods [57].

Clutch size (CS), defined as the number of eggs per sac, was relatively stable across different regions in summer, ranging from 18 to 27 eggs, with a mean of 21 to 24 eggs (Table 2). Maximum clutch sizes were observed in the coastal waters (MCW, NZW, and PCW) [73]. In autumn 2007, CS varied from 16 to 32 eggs per sac (Table 2).

Egg production rates (EPR) during the summer ranged from 0.09 to 1.84 eggs per female per day, with the highest rates occurring in MCW and AW [73]. A significant decline in mean EPR was evident from the southern to the northern Barents Sea (Table 2). Interestingly, EPR in autumn was higher than in summer, varying between 0.9 and 1.89 eggs per female per day [57]. Specific egg production rates (SEPR) for *O. similis* ranged from 0.002 to 0.045 day^−1^ during summer, with peak values observed in MCW and AW [73]. Mean SEPR was comparable in PCW, NZW, and ArW, while BSW showed intermediate values (Table 2). In autumn, SEPR values in MCW exceeded those recorded in the summer [58].

According to ANOVA and multiple comparisons, significant differences in the reproductive characteristics of *O. similis* were identified across the Barents Sea during summer (Table 3). The most pronounced differences were observed between the Arctic (ArW, BSW) and Atlantic (AW, MCW) regions [73]. Coastal areas also differed significantly from open sea sites (Table 3).

The relative abundance of ovigerous females of *Oithona similis* showed no significant correlations with fluctuations in environmental factors [57,73]. However, female FRE, CS, EPR, and SEPR were positively correlated with average water temperature, while a negative relationship was found with egg diameter [73]. An increase in the proportion of ovigerous females and egg diameter was associated with higher salinity levels, whereas FRE decreased with increasing salinity. Overall, temperature and salinity accounted for 50–93% of the total variance in female reproductive parameters. Additionally, Chl-a positively impacted CS and SEPR in *O. similis* [73].

Reproductive patterns of *Oithona* spp. have been studied in other Arctic and sub-Arctic regions. In Greenland waters, ovigerous females ranged from 30% to 80% from March to August 2010, with a relative abundance of 12% to 56% observed during the winter-spring period [51]. The mean clutch size for *Oithona* spp. was reported to be 20–27 eggs per female, which positively correlated with temperature during the summer [52]. In contrast, the mean clutch size during winter-spring ranged from 14 to 38, peaking during the post-bloom period [51]. Mean EPRs were recorded as 0.09–0.30, 0.09–0.91, and 0.13–1.65 eggs per female per day during winter, spring, and summer, respectively [51,52]. The mean SEPR was highest at 0.038 day^−1^ in summer, while winter and spring values did not exceed 0.019 day^−1^ [51]. Both EPR and clutch size showed positive correlations with water temperature, but no significant correlations with Chl-a during the summer. EPR increased significantly with protozooplankton biomass during the spring period [52]. In Balsfjord, Norway, mean clutch sizes of *O. similis* were found to be 8–9 in spring and 17–23 in summer during 2017–2018 under experimental conditions. The SEPR of *O. similis* varied from 0.02 day^−1^ in spring to 0.12 day^−1^ in summer and correlated with surface temperature [111].

Consequently, the reproduction of *O. similis* in the Barents Sea and adjacent Arctic regions is primarily influenced by fluctuations in water temperature. Furthermore, temperature has been identified as a key factor driving fecundity in *Oithona* spp. across other polar regions [45,49]. Experimental studies have indicated that EPR in *Oithona* is related to egg size, salinity, and temperature in various Arctic regions [41]. Food concentration also affects reproductive parameters, with protozooplankton density having a more significant impact than phytoplankton biomass, particularly in Arctic waters. This trend appears to be even more pronounced in temperate waters such as the North Sea and North Atlantic Ocean [42,54,55,69]. Recent investigations utilizing DNA barcoding have highlighted the dietary plasticity of *Oithona similis* in the Barents Sea. Phytoplankton constituted a significant portion of their winter-spring diet (up to 43%), while animal food items were more prevalent in other seasons [112]. As omnivorous organisms, *Oithona* spp. can sustain their activity year-round and are capable of reproducing throughout all seasons, as they are not limited by seasonal fluctuations in phytoplankton density. *Oithona* spp. play a crucial role as important copepods during winter and autumn when larger calanoid copepods are in diapause [38,68]. Therefore, *O. similis* is vital for high-Arctic ecosystems during periods of low activity and density of herbivorous zooplankton.

## 4. Mortality of *Oithona similis*

Instantaneous mortality rates for the stage pairs copepodite IV to copepodite V (MC4-C5) and copepodite V to adult (MC5-A) of *Oithona similis* were examined using a vertical life table approach [78,113,114]. The stage-specific daily mortality rates exhibited significant variations across different water masses, with distinct spatial patterns observed during periods of population peaks (Table 4).

Overall, the mortality rate for the stage pair copepodite IV to copepodite V (MC4-C5) varied from 0.003 to 0.335 day^−1^ during the summer period [115]. The highest rates were recorded at certain coastal sites within MCW and PCW (Table 4). In contrast, the minimum MC4-C5 rates were found in northern areas (ArW), ranging from 0.03 to 0.12 day^−1^, with low mean estimates (Table 4). Analysis of MC4-C5 dynamics in MCW revealed significant seasonal differences, with higher mortality observed in autumn (ANOVA, *p* < 0.05) (Table 4).

Spatial variations in the mortality of *O. similis*, particularly for copepodite V to adult (MC5-A), were documented in the Barents Sea. Maximum summer mortality rates were recorded in MCW at 0.382 day^−1^, while minimum levels were observed in ArW at 0.001 day^−1^ [57,115]. Mean values in AW, BSW, NZW, and PCW were similar during the summer period (Table 4). Regionally, mortality rates were generally higher in MCW compared to other water masses during the summer population growth phase, with C5-A summer mortality rates lower than those observed in autumn in MCW (Table 4).

The primary factors influencing mortality rates of *O. similis* in the Barents Sea are water temperature and salinity [78,115]. Temperature plays a significant role in high-salinity regions (ArW, AW, BSW), while salinity appears crucial in estuarine regions (MCW and PCW). A pronounced increase in mortality rates was associated with warm water areas (MCW), and there was a clear trend of decreasing mortality rates moving northwards. Salinity was negatively correlated with mortality rates, suggesting lower survival potential for *O. similis* in areas with reduced salinity, such as the Pechora River Delta and the mouth of Kola Bay [57,78,115]. Seasonal variations were also noted in Kola Bay. Here, MC4-C5 mortality rates were recorded as 0.005, 0.004, 0.004, and 0.002 day^−1^ for winter, spring, summer, and autumn, respectively. For MC5-A, the mortality rates were 0.058, 0.079, 0.228, and 0.120 day^−1^ for females, and 0.162, 0.207, 0.453, and 0.288 day^−1^ for males [57,115]. Thus, summer mortality rates for MC5-A were 2–3 times higher than in other seasons. In Kola Bay, no significant differences in MC4-C5 mortality rates were found among the four seasons, although winter estimates were higher than in other seasons. Overall, *O. similis* reached peak mortality levels in the summer in Kola Bay (Table 2). These values surpass mortality estimates from Arctic locations (e.g., Disco Bay, MC5-A at 0.003 day^−1^ [116]), Antarctic regions (e.g., Scotia Sea with MC4-C5 at 0.008 day^−1^, MC5-A females at 0.018 day^−1^, and males at 0.110 day^−1^ [114]), and temperate waters (e.g., North Sea, MC5-A females at 0.003 day^−1^ [113]). This variation is likely due to inter-annual differences in the structure of local pelagic communities, from microplankton to ichthyoplankton, and variations in hydrological regimes.

Water temperature was identified as the most critical factor affecting mortality rates of *O. similis* in Kola Bay. Moreover, a positive relationship was observed between Chl-a concentrations and mortality rates in this fjord. High temperatures and phytoplankton biomass may create favorable conditions for *O. similis*, potentially leading to increased abundance. However, higher abundance can also result in increased mortality due to intraspecific competition for space and food resources, as well as heightened vulnerability to parasites and predators, especially in favorable seasons [19,57,58,78,117]. Nevertheless, no direct relationships between mortality rates and Chl-a concentrations were found. Being a typical marine species, *O. similis* is adversely affected by low salinity levels, which can significantly contribute to its mortality, particularly in PCW.

In other Arctic regions, mortality rates also exhibited strong spatial and temporal fluctuations. For instance, in the White Sea in July 2001, mean summer MC4-C5 and MC5-A mortality rates for *O. similis* were 0.194 day^−1^ and 0.127 day^−1^, respectively [115]. In the northern part of the White Sea in July 2008, MC4-C5 mortality averaged 0.034 day^−1^, while MC5-A values ranged from 0.040 to 0.207 day^−1^, yielding a mean of 0.095 day^−1^. Similarly to the Barents Sea, the overall mortality of *O. similis* was positively correlated with water temperature and negatively correlated with salinity. In Disco Bay (Greenland waters), mortality rates were comparatively low (<0.06 day^−1^) across all developmental stages of *O. similis* in June 2001 [116].

In summary, the mortality of *O. similis* fluctuates significantly in the Barents Sea and other Arctic regions, with peak values generally associated with nutrient-rich, warm waters or freshened environments. Recent warming trends in the Arctic may be influencing the mortality patterns of *O. similis*, potentially impacting copepod assemblages in the region.

## 5. Morphological Variability and Populations of *Oithona similis*

In the southern Barents Sea (MCW), the modal prosome length (PL) is calculated to be 405 µm for males, and 450 µm and 465 µm for females. In the southeastern part (PCW), specimens with prosome lengths of 375 µm and 405 µm were observed in males, with females reaching up to 480 µm. In the central (AW, BSW) and eastern (NZW) regions, males exhibited a prosome length of 420 µm, while females were found in the 495 µm and 510 µm size classes [57,72]. In the northern and northeastern areas (ArW), the modal PL for males was found to be 465 µm and for females, it was 510 µm. Overall, the average prosome length of both sexes increased from south to north (Table 5).

Clear latitudinal trends in antennule length, number of setae, and relative antennule length were observed in both male and female *O. similis*, with each parameter decreasing as latitude increased (Table 5). The highest values were recorded in the southern regions, while the smallest were found in the northern areas.

In the Barents Sea, salinity and Chl-a explained very little of the variance in the morphological parameters of *O. similis* [57,72]. In contrast, the number of setae, absolute setae length, and both absolute and relative antennule lengths were positively correlated with temperature. Conversely, body size and relative setae lengths were negatively correlated with this environmental variable. At high latitudes, animals tend to grow more slowly, live longer, exhibit larger sizes, and have lower reproductive rates compared to their counterparts at lower latitudes [118,119,120]. An increase in cell size in a low-temperature environment is considered a general biological mechanism affecting body size in marine crustaceans [121]. Spatial variations in absolute and relative antennule and setae lengths reflect the adaptations of *O. similis* to different environmental conditions. A higher relative surface area of appendages and other morphological structures at lower latitudes—where water density is lower than at high latitudes—is crucial for maintaining neutral buoyancy [57,58].

**Figure 2:**
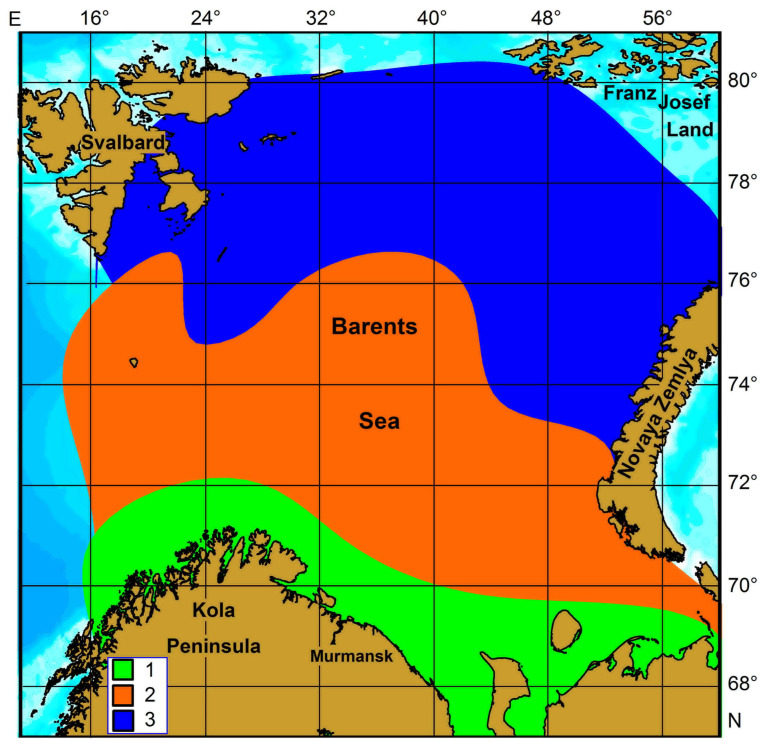
Population structure of *Oithona similis* in the Barents Sea [58]. Populations were delineated based on the morphology and reproductive features: 1—southern, 2—central, 3—northern.

Populations of *O. similis* were delineated using non-metric cluster analysis based on Euclidean distances between log10-transformed reproductive parameters, as well as discriminant analyses on the morphological parameters of these copepods [57,72]. Both methods identified three distinct populations (Figure 2).

Population 1, which includes *O. similis* individuals from the southern and central regions, corresponds to AW and MCW. This population shows an intermediate female prosome modal size of 480 µm, situated between the smaller boreal form and the larger Arctic form [57,72,73]. Population 2 is composed of individuals from the eastern part of the Barents Sea, associated with NZW and BSW. This population represents a transition between northern and southern sector populations [38]. Population 3 occurs in the northern Barents Sea and consists of larger *O. similis* individuals, with a modal female prosome length of 510 µm, from the ArW zone. This population aligns closely with the Arctic–Okhotsk Sea group described by Shuvalov [38].

A phylogeographic study of *O. similis* s.l. populations from the Arctic Ocean, the Southern Ocean and its northern boundaries, the North Atlantic, and the Mediterranean Sea—based on two gene fragments—revealed seven distinct, geographically delineated mitochondrial lineages, with divergences among lineages ranging from 8% to 24% [122]. In the Arctic Ocean, only one lineage of *O. similis* was identified, indicating low genetic variability of the species in this region [122]. Another study examined seven populations of *O. similis* in the Arctic and North Atlantic Oceans to investigate allele expression variation in natural populations [123]. The authors found low genomic differentiation across all populations studied. Thus, the three morphological groups, distinguished by their morphology and reproductive traits, can be viewed as local populations adapted to the specific environmental conditions of the primary water masses in the Barents Sea (coastal, Arctic, and Atlantic).

## 6. Life Cycles of *Oithona similis*

The life cycle of *Oithona similis* exhibits specific characteristics across different populations in the Barents Sea, particularly studied in Kola Bay (southern Barents Sea, MCW). In early winter, adults comprised 52% of the population, while late copepodites (CIV and CV) accounted for 48%, with nauplii absent [57,74]. As winter progressed, the relative abundance of adults decreased to 32%, and the late-stage copepodites increased to 68%. By the end of winter, CIV and CV dominated the population (80%). In spring, the abundance of adults rose to 71%, while CV numbers decreased by 9%, and CIV were absent in mid-May. Mass reproduction occurred in June, resulting in a high abundance of nauplii (up to 97%). By late June, all copepodite stages were present, though nauplii remained the most numerous (95%). In the latter half of the summer, nauplii numbers decreased from 87% to 49%, while the proportion of older copepodites and adults increased by 25% and 26%, respectively [57,73,74]. Mass spawning of a new generation was noted in September, with all stages represented in samples, including a majority of nauplii (70–73%). By early October, naupliar abundance dropped to 7%, CIV and CV reached 36%, and adults made up 57% of the population. By the end of autumn, the population comprised 60% adult males and females, and 40% older copepodites [57,58,73,74].

Seasonal variations in PL and reproductive characteristics of *O. similis* were documented throughout the year in Kola Bay [74]. The average sizes for CV and adults peaked in May. Female PL decreased by August but rose again by December. Males were the smallest in September, with their PL increasing in November and remaining stable during winter. Similar PL variability patterns were observed in CV. The species reproduces year-round in Kola Bay, with ovigerous females found across all seasons and two major peaks in July (39%) and September (28%). Mean clutch size varied from 20 to 26 eggs per female, peaking in June, September, and November. The mean egg diameter was highest in May and smallest during July–August. EPR and SEPR were lowest in May and between October and December (<0.04 eggs female^−1^ day^−1^ and <0.001 day^−1^), while maximum values reached 2.2 and 1.2 eggs female^−1^ day^−1^ (0.046 and 0.023 day^−1^) in July (autumn generation) and September (summer generation), respectively [57,73,74]. Environmental factors significantly influenced the population characteristics of *O. similis* in Kola Bay. Mean abundance of all stages (with the exception of CIV and CV) increased with water temperature. Adult and stage-CV copepodites’ PLs were negatively correlated with water temperature and positively correlated with salinity. Abundances exhibited negative correlations with salinity, except for CV. The proportion of ovigerous females, EPR, and SEPR increased significantly with rising water temperature and decreasing salinity [73,74].

**Figure 3:**
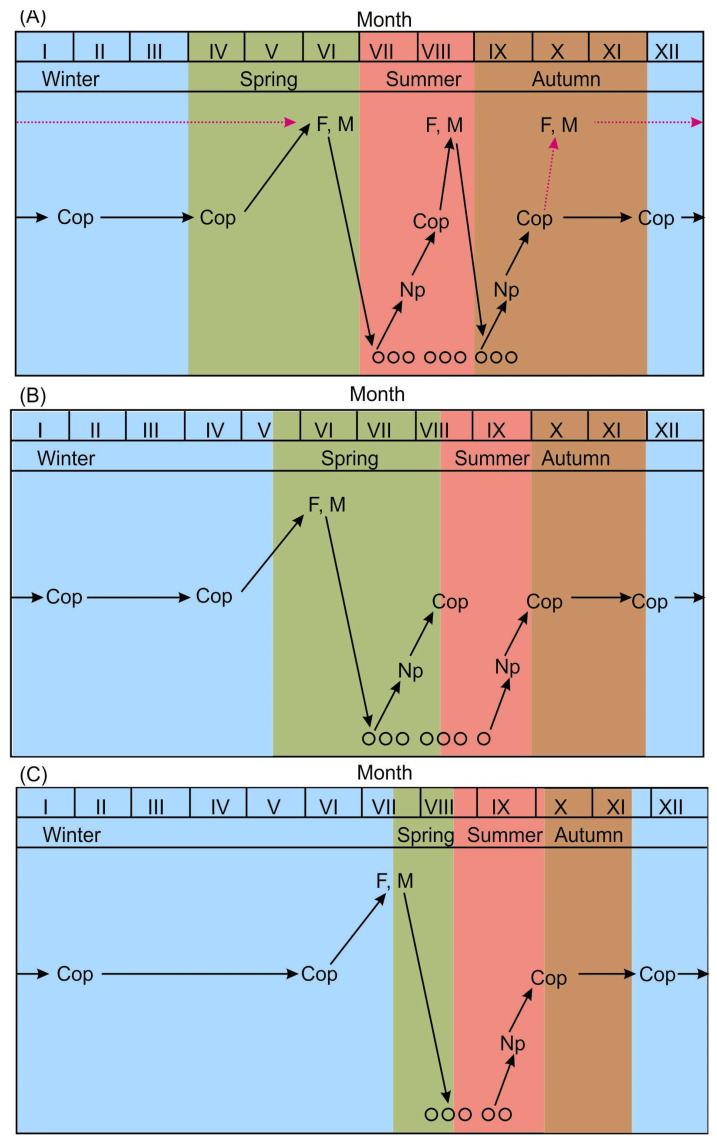
Schematic diagrams showing the life cycling of *Oithona similis* in the Barents Sea [57,58,73,74]. (**A**)—southern part (MCW), (**B**)—central part (AW and BSW), (**C**)—northern part (ArW). Black arrows indicate main reproduction/generations, dotted arrows indicate continuous reproducing throughout the year. Cop—copepodites, Np—nauplii, F—Females, M—Males, ooo—ova.

In summary, in the southern Barents Sea (MCW), *O. similis* abundance is low from December to May, predominantly consisting of older copepodites and few adults [124]. By late June, adult male and female abundances increase due to molting of V-stage copepodites from the previous year. Mass reproduction occurs at the end of June, characterized by high numbers of females with egg sacs, nauplii, and young copepodites. By July, all developmental stages are present, and the reproduction period concludes in late September, with males nearly absent outside of summer. Mass molting of nauplii into young copepodites occurs in late September, allowing these copepodites to overwinter until May. A small portion may continue to develop into adults by November, capable of reproducing during winter months (March–April) [57,74]. Thus, the life cycle of *O. similis* in this region spans 11–12 months (Figure 3A).

In the central Barents Sea (AW and BSW), there is a noticeable transition from overwintering CIV stages to CV stages at the beginning of May. Shortly after, the first adult specimens appear as spawning occurs in June, leading to a peak in reproducing females in July. Nauplii reach their peak abundance in early August, and by the end of September, they develop into CI–CIII and subsequently CIV–CV stages, which dominate the population from November to May. A small number of these stages may continue developing into adults during winter [57]. The life cycle of *O. similis* in this region lasts approximately 9–10 months (Figure 3B).

In the northern Barents Sea (ArW), developmental stages show strong seasonality. A shift from younger to older copepodites and adults is evident by the beginning of July. First egg-bearing females are observed at the end of July, with mass spawning occurring in early August. By mid-August, the population shifts to a predominance of nauplii, which transition into young copepodites of the next generation by October. Most of these individuals overwinter and do not spawn until August of the following year, although some copepodites may develop into adults and spawn in winter [58,72,73]. Thus, in Arctic waters, the life cycle lasts about 11 months (Figure 3C).

Across other Arctic regions, the life cycle of *O. similis* generally follows a pattern similar to that in the Barents Sea, with variations primarily in spawning times and abundance peaks. For example, in Kongsfjorden (northwestern Svalbard waters), *O. similis* reproduces year-round, with key reproductive periods in May–June and August–September [63]. Maximum abundance occurs in November, while minimum abundance is in June, possibly due to colder temperatures compared to Kola Bay. In Kola Bay, the life cycle of *O. similis* resembles that of the White Sea, where mass spawning occurs in May–June and July–August [57]. Developmental times for the first and second generations in the White Sea are 2 and 9–10 months, respectively [125]. Madsen et al. [65] reported that all life stages of *Oithona* spp. are present year-round in Disco Bay, with female populations comprising 40% during spring and summer. Two reproductive peaks have also been noted in western Greenland waters (June and August–September) [126]. In Godthåbsfjord (Greenland waters), adults and late copepodites are most abundant during winter and early spring, with stage composition shifting in May–June as younger stages emerge. Adult females of *Oithona* spp. constituted 20–83% of the population from April to June but decreased to 25% in summer when younger stages dominated. Adult males peaked in April–May (10–22%), while CIV–V stages exhibited peaks in April (15–58%) and July (13–40%). Nauplii were minimal (0–5%) in March–April but increased to 20–30% by early May, peaking at 50% in late May–June [52]. Contrarily, only one generation is present in the Greenland Sea [127], with few new generations maturing into adults. In Antarctic seas, *O. similis* also forms two generations annually [128], a pattern observed in Canadian Arctic waters [129] and northern Bering Sea regions [130]. However, only one generation is likely to occur in the Laptev Sea year-round [131].

## 7. Conclusions

*Oithona similis* demonstrates significant plasticity in body size, antennule length, and reproductive parameters (including egg diameter and production rates), allowing it to thrive across various water masses in the Barents Sea. This copepod prefers higher salinity and lower temperatures, displaying a seasonal life cycle with peaks in abundance and reproduction during the summer. In coastal areas affected by freshwater runoff, salinity is the primary factor influencing the abundance, reproductive rates, and mortality of *O. similis*. In contrast, temperature plays a more critical role at other locations. The relationship between temperature and population parameters can be both positive and negative, varying based on water mass characteristics, seasonal changes, and hydrological anomalies. Chlorophyll *a* concentrations appear less critical to *O. similis* than temperature and salinity. Further research is necessary to investigate the distribution patterns and seasonal abundance of late developmental stages of *O. similis*, utilizing appropriate sampling methods. Although prior studies in other regions indicate the significance of microplankton as a food source, there is limited information on microzooplankton prey and feeding behaviors specific to *O. similis* in the Barents Sea. Long-term studies have suggested the existence of three distinct populations of *O. similis*, but genetic research is essential to confirm this. Furthermore, routine monitoring of *O. similis* populations in the Barents Sea is critical given the ongoing shifts in climatic conditions in Arctic waters.

## Figures and Tables

**Table 1:** Abundance (individuals m^−3^), biomass (mg dry mass m^−3^), and relative biomass (contribution to the total zooplankton biomass, mean/min-max, %) of *Oithona similis* in the Barents Sea estimated from net sampling (Juday or WP2 nets, mesh size 168–180 μm). Water masses: MCW—Murmansk Coastal Water AW—Atlantic Water, ArW—Arctic Water, BSW—Barents Sea Water, PCW—Pechora Coastal Water, NZW—Novaya Zemlya Coastal Water.

Water Mass	Year	Season	Abundance	Biomass	Rel. Biomass	Reference
AW	2001	Summer	493	0.32	0.7/0.2–1.0	[90]
PCW	2001	Summer	100	0.07	0.9/0.0–5.8	[90]
MCW	2004	Summer	554	0.31	6.5/0.2–25.4	[57]
AW	2004	Summer	902	0.51	14.0/1.3–27.3	[57]
MCW	2005	Summer	951	0.61	6.5/2.6–12.6	[57]
AW	2005	Summer	664	0.43	1.3/1.2–1.4	[57]
MCW	2006	Summer	66	0.05	2.4/1.0–6.0	[73]
AW	2006	Summer	166	0.12	1.9/0.1–2.8	[73]
NZW	2006	Summer	73	0.06	3.6/0.2–9.5	[91]
ArW	2006	Summer	203	0.15	1.0/0.9–1.0	[92]
MCW	2007	Summer	1315	0.93	1.4/0.3–5.2	[73]
AW	2007	Summer	274	0.17	2.0/0.1–9.0	[73]
NZW	2007	Summer	1231	0.81	2.9/0.4–5.4	[73]
ArW	2007	Summer	814	0.47	3.8/1.3–6.4	[74]
MCW	2007	Autumn	366	0.26	2.2/2.2–6.4	[58]
MCW	2007	Winter	19	0.01	9.4/3.4–15.3	[58]
MCW	2007	Spring	331	0.23	1.1/0.1–3.6	[58]
MCW	2008	Summer	1650	1.20	5.0/1.3–11.7	[93]
MCW	2009	Summer	231	0.54	8.0/0.2–16.9	[77]
AW	2009	Summer	835	1.77	6.5/0.3–16.0	[77]
ArW	2009	Summer	768	1.22	6.3/0.2–16.9	[77]
NZW	2010	Summer	782	0.81	1.0/0.1–2.3	[76]
PCW	2010	Winter	492	1.00	1.7/0.1–3.5	[94]
BSW	2010	Winter	140	0.12	13.2/2.8–35.4	[95]
MCW	2010	Summer	1060	2.7	3.0/0.6–6.2	[61]
AW	2010	Summer	814	1.9	2.5/0.1–5.0	[61]
ArW	2010	Summer	1203	2.7	5.1/1.4–8.8	[61]
NZW	2010	Summer	385	1.0	3.3/0.1–11.2	[61]
MCW	2011	Summer	1590	3.3	5.1/1.2–14.4	[81]
MCW	2011	Autumn	81	0.08	0.5/0.1–1.5	[80]
AW	2011	Autumn	339	0.26	0.4/0.2–0.6	[80]
ArW	2011	Autumn	1184	0.99	0.4/0.1–16.1	[80]
MCW	2012	Winter	53	0.06	1.6/1.4–1.8	[96]
AW	2012	Winter	107	0.11	0.3/0.0–0.6	[96]
ArW	2012	Winter	624	0.57	0.4/0.1–0.9	[96]
PCW	2012	Summer	2864	2.8	3.9/0.6–6.4	[60]
MCW	2013	Summer	88	0.08	3.0/0.5–5.6	[97]
AW	2013	Summer	256	0.26	0.3/0.1–0.6	[97]
BSW	2013	Summer	158	0.17	1/0.3–2.4	[97]
ArW	2013	Summer	190	0.21	5.8/0.7–16.1	[97]
ArW	2015	Winter	227	0.23	1.4/0.7–2.4	[98]
MCW	2013	Summer	88	0.09	0.3/0.1–0.6	[79]
AW	2013	Summer	257	0.26	1.0/0.3–2.4	[79]
BSW	2013	Summer	158	0.17	5.8/0.7–16.1	[79]
ArW	2013	Summer	191	0.21	1.4/0.7–2.4	[79]
MCW + ArW	2013	Summer	48	0.04 *	0.7/0.1–2.2	[99]
MCW + ArW	2014–2017	Summer	499–999	0.38–0.86 *	0.1	[99]
AW	2001–2014	Summer	245	0.7	0.5/0.4–0.6	[100]
BSW	2015	Summer	178	0.2	0.6/0.1–2.5	[62]
ArW	2015	Summer	272	0.3	0.7/0.3–1.6	[62]
NZCW	2016	Spring	106	0.13	1.4/0.4–2.5	[59]
BSW	2016	Spring	220	0.26	1.8/0.7–2.5	[59]
AW	2019	Winter	188	0.18	1.18	[89]
AW	2019	Summer	1039	1.35	3.04	[89]
ARW	2019	Winter	188	0.18	1.18	[89]
ARW	2019	Summer	944	0.63	1.1/0.1–2.5	[89]
AW	2021	Winter	150	0.14	9.05	[89]
AW	2021	Spring	45	0.06	1.42	[89]
AW	2021	Summer	557	0.88	2.48	[89]
ARW	2021	Winter	307	0.45	2.62/0.5–8.2	[89]
ARW	2021	Spring	213	0.49	3.17/1.7–6.3	[89]
ARW	2021	Summer	311	0.80	1.85/1.4–2.3	[89]
AW	2021	Winter	135	0.1	0.8/0.2–2.8	[101]
AW	2021	Summer	3400	-	-	[102]
ArW	2021	Summer	600	-	-	[102]
NZCW	2022	Summer	2089	2.6	7.3/0.8–18.2	[103]

Note: * recalculated assuming 1 mg wet mass = 0.2 mg dry mass [104].

**Table 2:** Reproduction characteristics (mean values) of *Oithona similis* in the Barents Sea [57,58,73,74]. Water masses: MCW—Murmansk Coastal Water, AW—Atlantic Water, ArW—Arctic Water, BSW—Barents Sea Water, PCW—Pechora Coastal Water, NZW—Novaya Zemlya Coastal Water. OF—proportion of ovigerous females (%), D—egg diameter (μm), Fec—fecundity (eggs per sacs), RE—reproductive effort (%), EPR—egg production rate (eggs per female day^−1^), SEPR—specific egg production rate (day^−1^).

Water Mass	Year	Season	OF	RE	D	Fec	EPR	SEPR
AW	2001	Summer	33.7	33.0	63.0	23.0	0.88	0.026
PCW	2001	Summer	28.6	35.1	48.7	23.9	0.68	0.010
MCW	2004	Summer	21.9	39.2	48.7	25.8	0.91	0.012
AW	2004	Summer	15.3	37.7	50.4	25.3	0.41	0.006
MCW	2005	Summer	36.5	44.2	64.3	20.8	1.11	0.033
AW	2005	Summer	22.9	44.8	65.3	21.2	0.55	0.017
BSW	2005	Summer	12.3	44.2	64.4	20.8	0.19	0.006
MCW	2006	Summer	30.3	32.7	50.0	22.3	1.28	0.019
AW	2006	Summer	31.3	31.3	50.5	22.0	1.10	0.017
BSW	2006	Summer	30.5	29.8	56.3	21.4	0.72	0.015
NZW	2006	Summer	31.1	29.9	51.7	22.0	0.57	0.009
ArW	2006	Summer	31.5	26.9	67.6	21.0	0.33	0.011
MCW	2007	Summer	31.7	28.0	52.2	20.6	1.34	0.021
AW	2007	Summer	49.5	25.2	57.8	18.6	1.58	0.034
BSW	2007	Summer	47.0	29.9	57.3	21.7	1.43	0.030
NZW	2007	Summer	35.8	30.0	53.8	22.1	0.64	0.011
ArW	2007	Summer	35.3	27.0	68.0	21.3	0.31	0.010
MCW	2007	Autumn	31.3	34.2	55.3	23.1	1.35	0.028
MCW	2008	Summer	33.0	33.6	59.6	23.1	1.24	0.031

**Table 3:** Comparisons of reproduction characteristics (mean values) of *Oithona similis* in the Barents Sea [57,58,73]. Water masses: MCW—Murmansk Coastal Water AW—Atlantic Water, ArW—Arctic Water, BSW—Barents Sea Water, PCW—Pechora Coastal Water, NZW—Novaya Zemlya Coastal Water. OF—proportion of ovigerous females (%), D—egg diameter (μm), Fec—fecundity (eggs per sacs), RE—reproductive effort (%), EPR—egg production rate (eggs per female day^−1^), SEPR—specific egg production rate (day^−1^).

Comparisons		Reproductive Parameters					
		OF	D	Fec	RE	EPR	SEPR
Kruskal–Wallis Test	H	10.5	71.2	42.8	70	80.9	61.5
	*p*	0.06	<0.001	<0.001	<0.001	<0.001	<0.001
Pairs of water mass with significant differences	BSW-PCW, MCW, AW, NZW, ArW	PCW-AW, MCW, BSW, ArW	BSW-PCW	ArW-AW, PCW, MCW, BSW	ArW-PCW, AW, MCW	NZW-AW, MCW	BSW-PCW, MCW, AW, NZW, ArW
	PCW-BSW	NZW-BSW, ArW	ArW-MCW, PCW	NZW-PCW, MCW, BSW	BSW-AW, MCW	PCW-AW, MCW	PCW-BSW
Tukey–Kramer Test	MCW-BSW	AW-PCW, BSW, ArW	NZW-	AW-ArW, BSW	NZW-AW, MCW	ArW-AW, MCW	MCW-BSW
*p* < 0.05	AW-BSW	MCW-PCW, BSW, ArW	AW-PCW	PCW-ArW, NZW	PCW-ArW, AW, MCW	BSW-MCW	AW-BSW
	NZW-BSW	BSW-PCW, NZW, AW, MCW, ArW	MCW-ArW	MCW-ArW, NZW	AW-ArW, BSW, NZW, PCW	AW-NZW, PCW, ArW, MCW	NZW-BSW
	ArW-BSW	ArW-PCW, NZW, AW, MCW, BSW	PCW-BSW, ArW, AW	BSW-ArW, NZW, AW	MCW-ArW, BSW, NZW, PCW	MCW-NZW, PCW, ArW, BSW, AW	ArW-BSW
	BSW-PCW, MCW, AW, NZW, ArW	PCW-AW, MCW, BSW, ArW	BSW-PCW	ArW-AW, PCW, MCW, BSW	ArW-PCW, AW, MCW	NZW-AW, MCW	BSW-PCW, MCW, AW, NZW, ArW

**Table 4:** Mean mortality rates (day^−1^) of *Oithona similis* in the Barents Sea [57,58,78].

Water Mass	Year	Season	Pair	
			Copepodite IV-V	Copepodite V-Adults
AW	2001	Summer	0.011	0.113
PCW	2001	Summer	0.113	0.056
MCW	2004	Summer	0.044	0.105
AW	2004	Summer	0.024	0.060
MCW	2005	Summer	0.078	0.088
AW	2005	Summer	0.005	0.084
BSW	2005	Summer	0.006	0.073
MCW	2006	Summer	0.098	0.070
AW	2006	Summer	0.067	0.072
BSW	2006	Summer	0.076	0.022
NZW	2006	Summer	0.030	0.012
ArW	2006	Summer	0.012	0.045
MCW	2007	Summer	0.062	0.319
AW	2007	Summer	0.061	0.114
BSW	2007	Summer	0.075	0.214
NZW	2007	Summer	0.086	0.140
ArW	2007	Summer	0.050	0.030
MCW	2007	Autumn	0.107	0.206
MCW	2008	Summer	0.067	0.151
MCW	2009	Summer	0.218	0.005
AW	2009	Summer	0.135	0.001
ArW	2009	Summer	0.054	0.001
MCW (Kola Bay)	2005	Winter	0.005	0.087
MCW (Kola Bay)	2005	Spring	0.004	0.109
MCW (Kola Bay)	2006	Summer	0.004	0.300
MCW (Kola Bay)	2005	Autumn	0.002	0.154

**Table 5:** Latitudinal trends in morphological variability of *Oithona similis* in the Barents Sea (modified from [57,58,72]).

Parameter	Water Mass							
	MCW		PCW		AW		ArW	
	X ± SD	Range	X ± SD	Range	X ± SD	Range	X ± SD	Range
Male								
La, µm	925 ± 34	868–980	883 ± 28	840–952	811 ± 46	756–952	799 ± 26	756–868
Ns	36 ± 3	30–40	36 ± 3	30–40	34 ± 3	28–38	34 ± 2	28–36
Ls, µm	68 ± 2	64–72	67 ± 2	64–71	65 ± 4	59–70	64 ± 3	59–69
La/Lc, %	223 ± 3	217–231	231 ± 5	222–245	190 ± 7	171–204	177 ± 5	155–188
Ls/La, %	7.0 ± 0.3	6.6–7.5	7.3 ± 0.2	6.9–7.6	7.9 ± 0.4	6.8–8.4	8.1 ± 0.2	7.5–8.4
Female								
La, µm	863 ± 39	812–924	830 ± 30	784–896	806 ± 48	728–896	783 ± 30	700–808
Ns	37 ± 3	32–40	36 ± 3	30–40	34.2 ± 3.1	30–40	34 ± 2	30–36
Ls, µm	65 ± 1	64–69	65 ± 1	64–66	64 ± 1	64–65	65 ± 1	64–66
La/Lc, %	183 ± 10	166–210	176 ± 10	156–200	160 ± 5	150–173	153 ± 6	140–164
Ls/La, %	7.9 ± 0.4	7.3–8.8	8.1 ± 0.2	7.7–8.5	8.1 ± 0.3	6.8–8.8	8.2 ± 0.4	7.5–9.0

Note. MCW—Murmansk Coastal Water, PCW—Pechora coastal waters, AW—Atlantic Water, ArW—Arctic Water, Lc—total cephalothorax length, La—total antennule length, Ns: total number of setae on both antennules, Ls—total setae length, La/Lc—relative antennule length, Ls/La—relative setae length.

## Data Availability

No new data were created or analyzed in this study. Data sharing is not applicable to this article.

## References

[1] Rudels B. (2021). The Physical Oceanography of the Arctic Mediterranean Sea: Explorations, Observations, Interpretations.

[2] Jakobsen T., Ozhigin V.K. (2011). The Barents Sea: Ecosystem, Resources, Management: Half a Century of Russian-Norwegian Cooperation.

[3] Loeng H., Drinkwater K. (2007). An overview of the ecosystems of the Barents and Norwegian Seas and their response to climate variability. Deep-Sea Res. II.

[4] Schauer U., Loeng H., Rudels B., Ozhigin V.K., Dieck W. (2002). Atlantic water flow through the Barents and Kara Seas. Deep-Sea Res. I.

[5] Carmack E., Barber D., Christensen J., Macdonald R., Rudels B., Sakshaug E. (2006). Climate variability and physical forcing of the food webs and the carbon budget on panarctic shelves. Prog. Oceanogr..

[6] Dvoretsky A.G., Dvoretsky V.G. (2026). Fluctuations of net primary production along a standard transect in the Barents Sea and their relationships with environmental factors. Environ. Res..

[7] Wassmann P., Reigstad M., Haug T., Rudels B., Carroll M.L., Hop H., Gabrielsen G.W., Falk-Petersen S., Denisenko S.G., Arashkevich E. (2006). Food webs and carbon flux in the Barents Sea. Prog. Oceanogr..

[8] Oziel L., Sirven J., Gascard J.C. (2016). The Barents Sea frontal zones and water masses variability (1980–2011). Ocean Sci..

[9] Sakshaug E. (1997). Biomass and productivity distributions and their variability in the Barents Sea. ICES J. Mar. Sci..

[10] Reigstad M., Carroll J., Slagstad D., Ellingsen I., Wassmann P. (2011). Intra-regional comparison of productivity, carbon flux and ecosystem composition within the northern Barents Sea. Prog. Oceanogr..

[11] Sakshaug E., Stein R., Macdonald R.W. (2004). Primary and secondary production in the Arctic Seas. The Organic Carbon Cycle in the Arctic Ocean.

[12] Sakshaug E., Bjørge A., Gulliksen B., Loeng H., Mehlum F. (1994). Structure, biomass distribution, and energetics of the pelagic ecosystem in the Barents Sea: A synopsis. Polar Biol..

[13] Sakshaug E., Johnsen G., Kovacs K. (2009). Ecosystem Barents Sea.

[14] ICES (2022). Working Group on the Integrated Assessments of the Barents Sea (WGIBAR). ICES Sci. Rep..

[15] Gjøsæter H. (1995). Pelagic fish and the ecological impact of the modern fishing industry in the Barents Sea. Arctic.

[16] Eide A. (2017). Climate change, fisheries management and fishing aptitude affecting spatial and temporal distributions of the Barents Sea cod fishery. Ambio.

[17] Loeng H. (1991). Features of the physical oceanographic conditions in the central parts of the Barents Sea. Polar Res..

[18] Ozhigin V.K., Ivshin V.A. (1999). Water Masses of the Barents Sea.

[19] Raymont J.E.G. (1983). Plankton and productivity of the Oceans, Volume 2: Zooplankton.

[20] Woodd-Walker R.S., Ward P., Clarke A. (2002). Large-scale patterns in diversity and community structure of surface water copepods from the Atlantic Ocean. Mar. Ecol. Prog. Ser..

[21] Cornils A., Sieger R., Mizdalski E., Schumacher S., Grobe H., Schnack-Schiel S.B. (2018). Copepod species abundance from the Southern Ocean and other regions (1980–2005)—A legacy. Earth Syst. Sci. Data.

[22] Hsieh C.H., Chen C.S., Chiu T.S. (2005). Composition and abundance of copepods and ichthyoplankton in Taiwan Strait (western North Pacific) are influenced by seasonal monsoons. Mar. Freshw. Res..

[23] Reynolds C.S. (2008). A changing paradigm of pelagic food webs. Int. Rev. Hydrobiol..

[24] Dalpadado P., Yamaguchi A., Ellertsen B., Johannessen S. (2008). Trophic interactions of macro-zooplankton (krill and amphipods) in the Marginal Ice Zone of the Barents Sea. Deep Sea Res. II.

[25] Bernal A., Olivar M.P., Maynou F., de Puelles M.L.F. (2015). Diet and feeding strategies of mesopelagic fishes in the western Mediterranean. Prog. Oceanogr..

[26] Armengol L., Franchy G., Ojeda A., Santana-del Pino Á., Hernández-León S. (2017). Effects of copepods on natural microplankton communities: Do they exert top-down control?. Mar. Biol..

[27] Mauchline J. (1998). The Biology of Calanoid Copepods.

[28] Schukat A., Auel H., Teuber L., Lahajnar N., Hagen W. (2014). Complex trophic interactions of calanoid copepods in the Benguela upwelling system. J. Sea Res..

[29] Aarflot J.M., Skjoldal H.R., Dalpadado P., Skern-Mauritzen M. (2018). Contribution of *Calanus* species to the mesozo plankton biomass in the Barents Sea. ICES J. Mar. Sci..

[30] Svensen C., Halvorsen E., Vernet M., Franzè G., Dmoch K., Lavrentyev P.J., Kwasniewski S. (2019). Zooplankton communities associated with new and regenerated primary production in the Atlantic inflow north of Svalbard. Front. Mar. Sci..

[31] Tande K.S. (1991). *Calanus* in North Norwegian fjords and in the Barents Sea. Polar Res..

[32] Falk-Petersen S., Pedersen G., Kwasniewski S., Hegseth E.N., Hop H. (1999). Spatial distribution and life-cycle timing of zooplankton in the marginal ice zone of the Barents Sea during the summer melt season in 1995. J. Plankton Res..

[33] Dalpadado P., Arrigo K.R., van Dijken G.L., Skjoldal H.R., Bagøien E., Dolgov A.V., Prokopchuk I.P., Sperfeld E. (2020). Climate effects on temporal and spatial dynamics of phytoplankton and zooplankton in the Barents Sea. Progr. Oceanogr..

[34] Gallienne C.P., Robins D.B. (2001). Is *Oithona* the most important copepod in the world’s oceans?. J. Plankton Res..

[35] Turner J.T. (2004). The importance of small planktonic copepods and their roles in pelagic marine food webs. Zool. Stud..

[36] Skjoldal H.R., Aarflot J.M. (2023). Abundance and biomass of copepods and cladocerans in Atlantic and Arctic domains of the Barents Sea ecosystem. J. Plankton Res..

[37] Fish C.J. (1936). The biology of *Oithona similis* in the Gulf of Maine and Bay of Fundy. Biol. Bull..

[38] Shuvalov V.S. (1980). Copepod Cyclopoids of the Family Oithonidae of the World Ocean.

[39] Nishida S. (1985). Taxonomy and distribution of the family Oithonidae (Copepoda, Cyclopoida) in the Pacific and Indian oceans. Bull. Ocean Res. Inst. Univ. Tokyo.

[40] Böttger-Schnack R., Schnack D., Weikert H. (1989). Biological observations on small cyclopoid copepods in the Red Sea. J. Plankton Res..

[41] Nielsen T.G., Møller E.F., Satapoomin S., Ringuette M., Hopcroft R.R. (2002). Egg hatching rate of the cyclopoid copepod *Oithona similis* in arctic and temperate waters. Mar. Ecol. Prog. Ser..

[42] Nielsen T.G., Sabatini M. (1996). Role of cyclopoid copepods *Oithona* spp. in North Sea plankton communities. Mar. Ecol. Prog. Ser..

[43] Hansen F.C., Mollmann C., Schutz U., Hinrichsen H.H. (2004). Spatio-temporal distribution of *Oithona similis* in the Bornholm Basin (Central Baltic Sea). J. Plankton Res..

[44] Castellani C., Irigoien X., Harris R.P., Holliday N.P. (2007). Regional and temporal variation of *Oithona* spp. biomass, stage structure and productivity in the Irminger Sea, North Atlantic. J. Plankton Res..

[45] Ward P., Hirst A.G. (2007). *Oithona similis* in a high latitude ecosystem: Abundance, distribution and temperature limitation of fecundity rates in a sac spawning copepod. Mar. Biol..

[46] Temperoni B., Viñas M.D., Diovisalvi N., Negri R. (2011). Seasonal production of *Oithona nana* Giesbrecht, 1893 (Copepoda: Cyclopoida) in temperate coastal waters off Argentina. J. Plankton Res..

[47] Dahms H.U., Tseng L.C., Hwang J.S. (2015). Biogeographic distribution of the cyclopoid copepod genus *Oithona*–from mesoscales to global scales. J. Exp. Mar. Biol. Ecol..

[48] Cornwell L.E., Findlay H.S., Fileman E.S., Smyth T.J., Hirst A.G., Bruun J., McEvoy A.J., Widdicombe C.E., Castellani C., Lewis C. (2018). Seasonality of *Oithona similis* and *Calanus helgolandicus* reproduction and abundance: Contrasting responses to environmental variation at a shelf site. J. Plankton Res..

[49] Metz C. (1995). Seasonal variation in the distribution and abundance of *Oithona* and *Oncaea* species (Copepoda, Crustacea) in the southeastern Weddell Sea, Antarctica. Polar Biol..

[50] Paffenhöfer G.A. (1993). On the ecology of marine cyclopoid copepods (Crustacea, Copepoda). J. Plankton Res..

[51] Zamora-Terol S., Nielsen T.G., Saiz E. (2013). Plankton community structure and role of *Oithona similis* on the western coast of Greenland during the winter-spring transition. Mar. Ecol. Prog. Ser..

[52] Zamora-Terol S., Kjellerup S., Swalethorp R., Saiz E., Nielsen T.G. (2014). Population dynamics and production of the small copepod *Oithona* spp. in a subarctic fjord of West Greenland. Polar Biol..

[53] Nakamura Y., Turner J.T. (1997). Predation and respiration by the small cyclopoid copepod *Oithona similis*: How important is feeding on ciliates and heterotrophic flagellates?. J. Plankton Res..

[54] Castellani C., Irigoien X., Harris R.P., Lampitt R.S. (2005). Feeding and egg production of *Oithona similis* in the North Atlantic. Mar. Ecol. Prog. Ser..

[55] Castellani C., Irigoien X., Mayor D.J., Harris R.P., Wilson D. (2008). Feeding of *Calanus finmarchicus* and *Oithona similis* on the microplankton assemblage in the Irminger Sea, North Atlantic. J. Plankton Res..

[56] Dvoretsky V.G., Dvoretsky A.G. (2010). Checklist of fauna found in zooplankton samples from the Barents Sea. Polar Biol..

[57] Dvoretsky V.G., Dvoretsky A.G. (2011). Biology and Role of Oithona similis in Zooplankton of Arctic Seas.

[58] Dvoretsky V.G., Dvoretsky A.G. (2015). Ecology of Zooplankton Communities in the Barents Sea and Adjacent Waters.

[59] Dvoretsky V.G., Dvoretsky A.G. (2022). Coastal mesozooplankton assemblages during spring bloom in the eastern Barents Sea. Biology.

[60] Dvoretsky V.G., Dvoretsky A.G. (2023). Copepod assemblages in a large Arctic coastal area: A baseline summer study. Diversity.

[61] Dvoretsky V.G., Dvoretsky A.G. (2024). Marine copepod assemblages in the Arctic: The effect of frontal zones on biomass and productivity. Mar. Environ. Res..

[62] Dvoretsky V.G., Dvoretsky A.G. (2024). Local variability of Arctic mesozooplankton biomass and production: A case summer study. Environ. Res..

[63] Lischka S., Hagen W. (2005). Life histories of the copepod *Pseudocalanus minutus*, *P. acuspes* (Calanoida), and *Oithona similis* (Cyclopoida) in the Arctic Kongsfjorden (Svalbard). Polar Biol..

[64] Lischka S., Hagen W. (2007). Seasonal lipid dynamics of the copepods *Pseudocalanus minutus* (Calanoida) and *Oithona similis* (Cyclopoida) in the Arctic Kongsfjorden (Svalbard). Mar. Biol..

[65] Madsen S.D., Nielsen T.G., Hansen B.W. (2008). Annual population development of small-sized copepods in Disko Bay. Mar. Biol..

[66] Svensen C., Seuthe L., Vasilyeva Y., Pasternak A., Hansen E. (2011). Zooplankton distribution across Fram Strait in autumn: Are small copepods and protozooplankton important?. Prog. Oceanogr..

[67] Barth-Jensen C., Daase M., Ormańczyk M.R., Varpe Ø., Kwaśniewski S., Svensen C. (2022). High abundances of small copepods early developmental stages and nauplii strengthen the perception of a non-dormant Arctic winter. Polar Biol..

[68] Degtereva A.A. (1979). The regularities in plankton quantitative development in the Barents Sea. Trudy PINRO.

[69] Sabatini M., Kiørboe T. (1994). Egg production, growth and development of the cyclopoid copepod *Oithona similis*. J. Plankton Res..

[70] Castellani C., Licandro P., Fileman E., di Capua I., Mazzocchi M.G. (2016). *Oithona similis* likes it cool: Evidence from two long-term time series. J. Plankton Res..

[71] Cornwell L.E., Fileman E.S., Bruun J.T., Hirst A.G., Tarran G.A., Findlay H.S., Lewis C., Smyth T.J., McEvoy A.J., Atkinson A. (2020). Resilience of the copepod *Oithona similis* to climatic variability: Egg Production, mortality, and vertical habitat partitioning. Front. Mar. Sci..

[72] Dvoretsky V.G., Dvoretsky A.G. (2009). Morphological plasticity in the small copepod *Oithona similis* in the Barents and White Seas. Mar. Ecol. Prog. Ser..

[73] Dvoretsky V.G., Dvoretsky A.G. (2009). Spatial variations in reproductive characteristics of the small copepod *Oithona similis* in the Barents Sea. Mar. Ecol. Prog. Ser..

[74] Dvoretsky V.G., Dvoretsky A.G. (2009). Life cycle of *Oithona similis* (Copepoda: Cyclopoida) in Kola Bay (Barents Sea). Mar. Biol..

[75] Dvoretsky V.G., Dvoretsky A.G. (2013). Epiplankton in the Barents Sea: Summer variations of mesozooplankton biomass, community structure and diversity. Cont. Shelf Res..

[76] Dvoretsky V.G., Dvoretsky A.G. (2013). Summer mesozooplankton community of Moller Bay (Novaya Zemlya Archipelago, Barents Sea). Oceanologia.

[77] Dvoretsky V.G., Dvoretsky A.G. (2013). Structure of mesozooplankton community in the Barents Sea and adjacent waters in August 2009. J. Nat. Hist..

[78] Dvoretsky V.G. (2012). Seasonal mortality rates of *Oithona similis* (Cyclopoida) in a large Arctic fjord. Polar Sci..

[79] Dvoretsky V.G., Dvoretsky A.G. (2025). Summer zooplankton assemblages in the Barents Sea: Spatial variations and effects of environmental conditions as revealed from in situ and satellite data. Prog. Oceanogr..

[80] Dvoretsky V.G., Dvoretsky A.G. (2025). Effects of water temperature on zooplankton abundance and biomass in the southwestern Barents Sea: Implications for Arctic monitoring and management. Ocean Coast. Manag..

[81] Dvoretsky V.G., Dvoretsky A.G. (2026). Coastal zooplankton assemblages in the southern Barents Sea: A summer pattern of diversity and production. Mar. Environ. Res..

[82] Wassmann P. (2011). Arctic marine ecosystems in an era of rapid climate change. Prog. Oceanogr..

[83] Koenigk T., Key J., Vihma T., Kokhanovsky A., Tomasi C. (2020). Climate Change in the Arctic. Physics and Chemistry of the Arctic Atmosphere.

[84] Ardyna M., Arrigo K.R. (2020). Phytoplankton dynamics in a changing Arctic Ocean. Nat. Clim. Change.

[85] Malik I.H., Ahmed R., Ford J.D., Hamidi A.R. (2025). Arctic warming: Cascading climate impacts and global consequences. Climate.

[86] Polyakov I.V., Alkire M.B., Bluhm B.A., Brown K.A., Carmack E.C., Chierici M., Danielson S.L., Ellingsen I., Ershova E.A., Gårdfeldt K. (2020). Borealization of the Arctic Ocean in response to anomalous advection from sub-arctic seas. Front. Mar. Sci..

[87] Lewis K.M., Van Dijken G.L., Arrigo K.R. (2020). Changes in phytoplankton concentration now drive increased Arctic Ocean primary production. Science.

[88] Dvoretsky V.G., Vodopianova V.V., Bulavina A.S. (2023). Effects of Climate Change on Chlorophyll *a* in the Barents Sea: A Long-Term Assessment. Biology.

[89] Wold A., Hop H., Svensen C., Søreide J.E., Assmann K.M., Ormanczyk M., Kwasniewski S. (2023). Atlantification influences zooplankton communities seasonally in the northern Barents Sea and Arctic Ocean. Prog. Oceanogr..

[90] Dvoretsky V.G., Dvoretsky A.G. (2009). Summer mesozooplankton structure in the Pechora Sea (south-eastern Barents Sea). Estuar. Coast. Shelf Sci..

[91] Dvoretsky V.G., Dvoretsky A.G. (2009). Summer mesozooplankton distribution near Novaya Zemlya (eastern Barents Sea). Polar Biol..

[92] Dvoretsky V.G., Dvoretsky A.G. (2011). Copepod communities off Franz Josef Land (northern Barents Sea) in late summer of 2006 and 2007. Polar Biol..

[93] Dvoretsky V.G., Dvoretsky A.G. (2010). Mesozooplankton structure in Dolgaya Bay (Barents Sea). Polar Biol..

[94] Dvoretsky V.G., Dvoretsky A.G. (2015). Early winter mesozooplankton of the coastal south-eastern Barents Sea. Estuar. Coast. Shelf Sci..

[95] Dvoretsky V.G., Dvoretsky A.G. (2018). Features of winter zooplankton assemblage in the Central Trough of the Barents Sea. Arct. Environ. Res..

[96] Dvoretsky V.G., Dvoretsky A.G. (2018). Mesozooplankton in the Kola Transect (Barents Sea): Autumn and winter structure. J. Sea Res..

[97] Dvoretsky V.G., Dvoretsky A.G. (2018). Latitudinal variations of zooplankton community structure and productivity in the Barents Sea (summer 2013). Arct. Antarct. Res..

[98] Dvoretsky V.G., Dvoretsky A.G. (2020). Arctic marine mesozooplankton at the beginning of the polar night: A case study for southern and south-western Svalbard waters. Polar Biol..

[99] Prokopchuk I.P., Trofimov A.G. (2019). Interannual dynamics of zooplankton in the Kola Section of the Barents Sea during the recent warming period. ICES J. Mar. Sci..

[100] Gluchowska M., Dalpadado P., Beszczynska-Möller A., Olszewska A., Ingvaldsen R.B., Kwasniewski S. (2017). Interannual zooplankton variability in the main pathways of the Atlantic water flow into the Arctic Ocean (Fram Strait and Barents Sea branches). ICES J. Mar. Sci..

[101] Dvoretsky V.G., Venger M.P., Vashchenko A.V., Vodopianova V.V., Pastukhov I.A., Maksimovskaya T.M. (2023). Marine Plankton during the Polar Night: Environmental Predictors of Spatial Variability. Biology.

[102] Gawinski C., Basedow S.L., Sundfjord A., Svensen C. (2024). Secondary production at the Barents Sea polar front in summer: Contribution of different size classes of mesozooplankton. Mar. Ecol. Prog. Ser..

[103] Dvoretsky V.G., Moiseev D.V., Venger M.P., Vashchenko A.V., Vodopianova V.V. (2025). Environmental control of Arctic marine zooplankton near a large archipelago during the summer season. Cont. Shelf Res..

[104] Postel L., Fock H., Hagen W., Harris R., Wiebe P., Lenz J., Skjoldal H.R., Huntley M. (2000). Biomass and abundance. ICES Zooplankton Methodology Manual.

[105] Dvoretsky V.G., Dvoretsky A.G. (2011). Mesozooplankton structure in the northern White Sea in July 2008. Polar Biol..

[106] Dvoretsky V.G., Dvoretsky A.G. (2009). Distribution of the under-ice mesozooplankton in the Kara Sea in February 2002. Polar Biol..

[107] Dvoretsky V.G., Dvoretsky A.G. (2015). Regional differences of mesozooplankton communities in the Kara Sea. Cont. Shelf Res..

[108] Balazy K., Boehnke R., Trudnowska E., Søreide J.E., Błachowiak-Samołyk K. (2021). Phenology of *Oithona similis* demonstrates that ecological flexibility may be a winning trait in the warming Arctic. Sci. Rep..

[109] Dvoretsky V.G., Dvoretsky A.G. (2017). Macrozooplankton of the Arctic—The Kara Sea in relation to environmental conditions. Estuar. Coast. Shelf Sci..

[110] Dvoretsky V.G., Dvoretsky A.G. (2019). Summer macrozooplankton assemblages of Arctic shelf: A latitudinal study. Cont. Shelf Res..

[111] Barth-Jensen C., Koski M., Varpe Ø., Glad P., Wangensteen O.S., Præbel K., Svensen C. (2020). Temperature-dependent egg production and egg hatching rates of small egg-carrying and broadcast-spawning copepods *Oithona similis*, *Microsetella norvegica* and *Microcalanus pusillus*. J. Plankton Res..

[112] Flo S., Svensen C., Præbel K., Bluhm B.A., Vader A. (2024). Dietary plasticity in small Arctic copepods as revealed with prey metabarcoding. J. Plankton Res..

[113] Eiane K., Ohman M.D. (2004). Stage-specific mortality of *Calanus finmarchicus*, *Pseudocalanus elongatus*, and *Oithona similis* on Fladen Ground, North Sea, during a spring bloom. Mar. Ecol. Prog. Ser..

[114] Hirst A.G., Ward P. (2008). Spring mortality of the cyclopoid copepod *Oithona similis* in polar waters. Mar. Ecol. Prog. Ser..

[115] Dvoretsky V.G., Dvoretsky A.G. (2011). The mortality of the planktonic copepod *Oithona similis* Claus, 1866 (Copepoda: Cyclopoida) in the Barents and White seas. Russ. J. Mar. Biol..

[116] Thor P., Nielsen T.G., Tiselius P. (2008). Mortality rates of epipelagic copepods in the post-spring bloom period in Disko Bay, western Greenland. Mar. Ecol. Prog. Ser..

[117] Viitasalo M., Kiørboe T., Flinkman J., Pedersen L.W., Visser A.W. (1998). Predation vulnerability of planktonic copepods: Consequences of predator foraging strategies and prey sensory abilities. Mar. Ecol. Prog. Ser..

[118] Clarke A. (1983). Life in cold water: The physiological ecology of polar marine ectotherms. Oceanogr. Mar. Biol. Annu. Rev..

[119] Clarke A. (1987). Temperature, latitude and reproductive effort. Mar. Ecol. Prog. Ser..

[120] Conover D.O. (1992). Seasonality and the scheduling of life history at different latitudes. J. Fish Biol..

[121] McLaren I.A., Sevigny J.M., Corkett C.J. (1988). Body size, development rates, and genome size among *Calanus* species. Hydrobiologia.

[122] Cornils A., Wend-Heckmann B., Held C. (2017). Global phylogeography of *Oithona similis* s.l. (Crustacea, Copepoda, Oithonidae)—A cosmopolitan plankton species or a complex of cryptic lineages?. Mol. Phylogenet. Evol..

[123] Laso-Jadart R., Sugier K., Petit E., Labadie K., Peterlongo P., Ambroise C., Wincker P., Jamet J.L., Madoui M.A. (2020). Investigating population-scale allelic differential expression in wild populations of *Oithona similis* (Cyclopoida, Claus, 1866). Ecol. Evol..

[124] Dvoretsky V.G., Dvoretsky A.G. (2012). Estimated copepod production rate and structure of mesozooplankton communities in the coastal Barents Sea during summer–autumn 2007. Polar Biol..

[125] Prygunkova R.V. (1974). Certain peculiarities in the seasonal development of zooplankton in the Chupa Inlet of the White Sea. Explor. Fauna Seas.

[126] Ussing H.H. (1938). The Biology of Some Important Plankton Animals in the Fjords of East Greenland.

[127] Digby P.S.B. (1954). The biology of marine plankton copepods of Scoresby Sound, East Greenland. J. Anim. Ecol..

[128] Atkinson A. (1998). Life cycle strategies of epipelagic copepods in the Southern Ocean. J. Mar. Syst..

[129] Hopcroft R.R., Clarke C., Nelson R.J., Raskoff K.A. (2005). Zooplankton communities of the Arctic’s Canada Basin: The contribution by smaller taxa. Polar Biol..

[130] Shaginyan E.R. (1982). Population dynamics and age structures of *Oithona similis* (Claus) and *Pseudocalanus elongatus* (Boeck) in the Bay of Oliyutorsk. Izv. TINRO.

[131] Lischka S., Knickmeier K., Hagen W. (2001). Mesozooplankton assemblages in the shallow Arctic Laptev Sea in summer 1993 and autumn 1995. Polar Biol..

